# Microbiological Characterization of the Biofilms Colonizing Bioplastics in Natural Marine Conditions: A Comparison between PHBV and PLA

**DOI:** 10.3390/microorganisms11061461

**Published:** 2023-05-31

**Authors:** Anna Marín, Patricia Feijoo, Rosa de Llanos, Belén Carbonetto, Pedro González-Torres, José Tena-Medialdea, José R. García-March, José Gámez-Pérez, Luis Cabedo

**Affiliations:** 1 Polymers and Advanced Materials Group (PIMA), Universitat Jaume I (UJI), Av. de Vicent Sos Baynat s/n, Castelló de la Plana, 12071 Castelló, Spain; anmarin@uji.es (A.M.); pfeijoo@uji.es (P.F.); gamez@uji.es (J.G.-P.); 2 MicroBIO, Universitat Jaume I (UJI), Av. de Vicent Sos Baynat s/n, Castelló de la Plana, 12071 Castelló, Spain; dellanos@uji.es; 3 Microomics Systems S.L., IIB Sant Pau, C/Sant Quintí, 77-79, 08041 Barcelona, Spain; belen.carbonetto@microomics.com (B.C.); pedro.gonzalez@microomics.com (P.G.-T.); 4 IMEDMAR-UCV Institute of Environment and Marine Science Research, Universidad Católica de Valencia, Av. del Port, 15, 03710 Calpe, Spain; josetena@ucv.es (J.T.-M.); jr.garcia@ucv.es (J.R.G.-M.)

**Keywords:** 16S rRNA, bacterial community, biodegradable plastics, biodegradation, seawater

## Abstract

Biodegradable polymers offer a potential solution to marine pollution caused by plastic waste. The marine biofilms that formed on the surfaces of poly(lactide acid) (PLA) and poly(3-hydroxybutyrate-co-3-hydroxyvalerate) (PHBV) were studied. Bioplastics were exposed for 6 months to marine conditions in the Mediterranean Sea, and the biofilms that formed on their surfaces were assessed. The presence of specific PLA and PHBV degraders was also studied. PHBV showed extensive areas with microbial accumulations and this led to higher microbial surface densities than PLA (4.75 vs. 5.16 log CFU/cm^2^). Both polymers’ surfaces showed a wide variety of microbial structures, including bacteria, fungi, unicellular algae and choanoflagellates. A high bacterial diversity was observed, with differences between the two polymers, particularly at the phylum level, with over 70% of bacteria affiliated to three phyla. Differences in metagenome functions were also detected, revealing a higher presence of proteins involved in PHBV biodegradation in PHBV biofilms. Four bacterial isolates belonging to the Proteobacteria class were identified as PHBV degraders, demonstrating the presence of species involved in the biodegradation of this polymer in seawater. No PLA degraders were detected, confirming its low biodegradability in marine environments. This was a pilot study to establish a baseline for further studies aimed at comprehending the marine biodegradation of biopolymers.

## 1. Introduction

Despite the efforts made by the international community to improve plastic waste disposal and sensitize society to the problems that their incorrect removal causes, a vast volume of residues enters the oceans each year (around 11 million tons) [1]. Accumulation of plastic wastes in the marine environment represents a global problem, affecting aquatic wildlife and human health [2,3]. In this context, the demand for biodegradable polymers is increasing, as they represent an alternative to non-biodegradable plastics that might help to partially mitigate these problems. They are particularly promising to counteract the current massive usage of single-use and short-shelf-life plastic products (e.g., food serving utensils and packaging). Thus, the production of biodegradable plastics is expected to rise from 1.2 to 1.8 million tons by 2025 and their potential fields of application to increasingly diversify [4]. However, the pre-assumed biodegradability of biopolymers can be misleading and result in inappropriate post-shelf-life management, which could increase their presence in the environment. One of the key issues is that, to be considered as biodegradable, a biopolymer only has to prove that it can biodegrade under a certain single condition, which does not ensure that this material does likewise under all conditions.

Poly(lactide acid) (PLA) and polyhydroxyalkanoates (PHAs) are considered the main contributors to the increase in biodegradable polymer demand [4,5,6]. These polymers possess thermoplastic processing capacity and, while PLA is obtained through the fermentation of starch as lactic acid, followed by chemical polymerization, PHAs are synthetized in vivo by microorganisms as intracellular carbon and energy storage [4,7,8,9]. PLA exhibits high mechanical strength but poor heat resistance and brittle fracture behavior, along with being a moderate barrier to gases and organic-compounds barrier [10,11]. On the other hand, PHAs display balanced mechanical properties in terms of stiffness and tensile strength but low toughness, together with relatively high water-vapor- and moderate oxygen-barrier properties [12,13]. Both can be categorized as bioplastics, since they are derived from renewable sources. However, their degradation behavior is significantly different. PHAs possess excellent biodegradation features that have been widely studied in different terrestrial environments [7], and they exhibit also good biodegradation in marine conditions, although the available literature is more limited and reported results quite inconsistent. For instance, [8] reported a weight loss of 36% in poly(3-hydroxybuyrate-co-3-hydroxyvalerate) (PHBV) samples after 180 days of exposure to natural marine conditions, while [14] observed that after 160 of exposure, the residual weight of different PHA samples was around 56%; and of 10% after 4 weeks in the case of [15]. PLA only biodegrades if temperature conditions are above its glass transition temperature (under industrial compositing), and its biodegradation is limited when exposed to natural and aquatic conditions [9,16]. As an example, biodegradation of PLA films was very low after immersion in seawater [17,18].

As the market for bioplastics grows and expands, their potential release into the environment becomes meaningful. In fact, an increase in PLA contamination in the environment has been observed in the last few years [9]. Consequently, an understanding of biopolymers’ biodegradation behavior in natural conditions, especially in the marine ecosystem, where degradation is considerably slower, is crucial [5,16]. Since biodegradation is a combination of abiotic and biotic phenomena leading to an ultimate material mineralization by microbial enzymes, the distribution, amount and type of the naturally-occurring microorganisms present on plastic surfaces (biofilm) are key factors in the overall process [8]. In the case of marine conditions, existent marine microbiota are strongly influenced by an interplay of abiotic factors, within which the type of polymer is a key factor [5,19]. Due to the essential contribution of the microbial biofilm covering plastic surfaces to the biodegradation process, this paper puts emphasis on the marine biofilms that form on PHBV and PLA films, to elucidate the microbiological aspects of their marine biodegradation and establish a baseline for further studies. It was expected that the different compositions and properties of the materials would lead to significant differences in the biofilm microbial compositions.

PHBV and PLA films were exposed to natural marine conditions in the western Mediterranean Sea for 6 months and the microbiota established on their surfaces were described and quantified through scanning electron microscopy (SEM), microbial counts and 16S rRNA gene amplicon sequencing. The biodegradation capabilities of different microbial isolates were also assessed.

## 2. Materials and Methods

### 2.1. Materials

PHBV commercial grade ENMAT Y100P (3 wt % valerate content, melting point 170–176 °C, density 1.23 g/cm^3^) and PLA commercial grade PLE 005 (melting point 175 °C, density 1.24 g/cm^3^) were supplied in pellet form by Tianan Biologic Material Co. (Ningbo, China) and NaturePlast (Ifs, France), respectively. Pellets were dried at 60 °C in a PIovan DPA 10 dehumidifying dryer (Santa Maria di Sala, VE, Italy) and extruded in a single-screw extruder (Haake Rheomex 252p, Karlsruhe, Germany) with a calendering system, to obtain film of 400 µm thickness. The temperature profile from hopper to fishtail nozzle was set at 140/165/175/170 °C for PHBV and at 160/170/180/190 °C for PLA and the rotation speed at 100 rpm.

### 2.2. Experimental Setup

**Figure 1:**
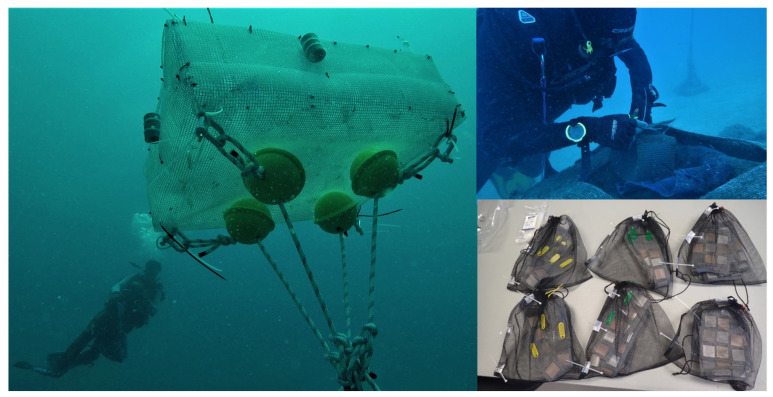
Submarine station sited in waters close to Calpe Port (Calpe, Spain) and experimental setup.

Thirteen film pieces (4 × 4 cm) of each material were cut, disinfected with ethanol and cleaned to remove impurities. Subsequently, they were placed inside custom-made fine meshed nylon bags which were introduced into semi-open cages. Bags were located in a submarine station sited in waters close to Calpe Port (38°37′54.4″ N 0°04′19.5″ E, Calpe, Spain) (Figure 1) and exposed during a 6-month period (September 2020 to March 2021). Two or three replicates of each material were retrieved monthly. After each extraction, nylon bags were transported to the Institute of Environment and Marine Science Research (Calpe, Spain) where they were stored at 4 °C and sent to the Polymers and Advanced Materials Laboratory (Castelló, Spain). Samples were analyzed within a maximum of 24 h after extraction.

### 2.3. Analysis of Biofilms by Scanning Electron Microscopy (SEM)

Qualitative composition and distribution of the biofilm formed on the film pieces’ surfaces were analyzed by SEM using a high-resolution field-emission JEOL 7001F microscope. Samples were coated by sputtering a thin layer of platinum and observed using an accelerating voltage of 5 kV. The observed microbial structures were compared with those described in previous works, and taxonomic classification, if possible, was based on morphological observations.

### 2.4. Quantification of Microbial Colonization and Dynamics

Each film piece was scraped with a sterile scalpel blade to ensure the maximum amount of biofilm was recovered. Each piece and the detached biofilm were placed in tubes containing 20 mL of phosphate-buffered saline (NaCl 8 g/L, KCl 0.2 g/L, Na_2_HPO_4_ 1.4 g/L, KH_2_PO_4_ 0.2 g/L) and vigorously shaken for 1 min. Biofilm suspensions were serially diluted by duplicate and spread on marine agar (MA) and potato dextrose agar with 50% of natural seawater (PDA) (Laboratorios Conda, S.A., Madrid, Spain) for the quantification of cultivable heterotrophic marine bacteria and marine yeasts and molds, respectively. Both media were supplemented either with cycloheximide (0.01 g/L) or streptomycin sulfate salt (0.05 g/L) (Merck Life Science S.L., Madrid, Spain) to suppress molds or bacteria growth, respectively. MA and PDA plates were incubated at 26 °C for 4 and 6 days and colony forming units (CFU) were counted. Results were expressed as log CFU/cm^2^ material.

### 2.5. Analysis of Biofilm Diversity and Composition by Amplicon Sequencing

#### 2.5.1. Amplicon Library Preparation and Sequencing

After 6 months of exposure, from 3 replicates per material, 5 circular pieces of 0.3 cm^2^ were cut with a hole puncher and placed in cryotubes containing DNA/RNA Shield stabilization solution (Zymo Research Corporation, Tustin, CA, USA). Samples were sent to Microomics Systems S.L. (Barcelona, Spain) where they were frozen at −80 °C until analyzed. DNA was extracted using the DNeasy PowerLyzer PowerSoil Kit (Qiagen, Hilden, Germany) following the manufacturer’s instructions. The extraction tubes were agitated using Tissue lyser II (Qiagen, Hilden, Germany) at 30 Hz/s for 10 min. Mock community DNA was included as positive control for library preparation (Zymobiomics Microbial Community DNA, ZymoResearch, Irvine, CA, USA) and to ensure quality control. Samples were amplified using 16S rRNA V3-V4 regions specific primers (V3-V4-Forward 5′-TCGTCGGCAGCGTCAGATGTGTATAAGAGACAGCCTACGGGNGGCWGCAG-3′, V3-V4 Reverse 5′GTCTCGTGGGCTCGGAGATGTGTATAAGAGACAGGACTACHVGGGTATCTAATCC-3′). Amplicon library preparation was performed as previously reported [20]. Negative controls included sample collection buffer, DNA extraction and PCR amplification blank. Sequencing was performed using an Illumina MiSeq (2 × 300 bp).

#### 2.5.2. Sequence Processing and Analysis

Raw, demultiplexed forward and reverse reads were processed using the methods and pipelines implemented in QIIME2 version 2020.11, with default parameters unless stated otherwise [21]. DADA2 was used for quality filtering, denoising, pair-end merging and amplicon sequence variant calling (ASV, i.e., phylotypes) using QIIME DADA2 denoise-paired method [22]. Details are provided in Appendix A. OTU tables were used to calculate unweighted and weighted Unifrac distances to compare community structures [23]. Alpha diversity metrics calculated were: observed operational taxonomic units (OTUs) number (i.e., richness) and Pielou`s evenness index. To assess the OTUs distribution in the biofilms, Venn diagrams were plotted using an online tool [24].

Potential functional profiles for sequenced samples were predicted using PICRUSt2 [20]. Phylotypes were placed into a reference tree containing 20,000 full 16S rRNA genes from prokaryotic genomes in the Integrated Microbial Genomes (IMG) database. Functional annotation of genomes was based on the Clusters of Orthologous Groups of proteins (COG) database. To infer the abundance of each gene family (i.e., COGs functions) per sample, the abundances of phylotypes were corrected by their 16S rRNA gene copy number and then multiplied by their functional predictions.

### 2.6. Screening of Biopolymer Degraders

For the detection of isolates able to specifically degrade the biopolymers, the clear zone method was used. This method involves inoculating the isolates onto agar containing the polymer. After incubation, the development of a clear zone around the colonies is considered an indication of polymer depolymerization [25,26]. MA and PDA plates incorporating the powdered biopolymers (20 g/L) were prepared. To this end, polymers were ground by cryogenic milling under liquid nitrogen and subsequent sieving through a 250 µm mesh. Different bacteria, yeasts and molds observed on microbial count plates corresponding to the last sampling time were selected and sub-cultured 3 times until pure cultures were obtained. Each isolated bacteria and yeast was inoculated in liquid medium and incubated at 26 °C for 48 h. Aliquots of 5 µL from each culture were deposited by duplicate on the corresponding plates. For fungi, 25 mm^2^ agar squares were cut from the outer edge of 5-day old active cultures using a sterile scalpel blade and placed on PDA. Plates were incubated at room temperature and observed after 5, 10 and 15 days to check the presence of clearance zones surrounding the colonies. Isolates which tested positive for biodegradation were individually re-inoculated on fresh medium and the appearance of clearing zones was monitored again. Different known bacterial strains were also tested following the same methodology [27,28,29]. Those showing degradative activity were used as positive controls in the screening experiment. Tested strains were acquired from the Spanish Type Collection (CECT, University of Valencia, Valencia, Spain).

### 2.7. Molecular Identification of Biopolymer Degraders

Bacterial isolates which tested positive (Section 2.6) were identified by means of the 16S rRNA marker gene. Briefly, “colony PCR” was performed using 27F and 1492R primers to amplify the 16S rRNA gene [30]. Amplicons were sequenced by Sanger sequencing. Control quality of sequences was done manually, and sequence ends were trimmed to avoid low quality bases. High quality sequences (600–800 bp length) were used as the query for a basic local alignment search tool (BLAST) search against the NCBI 16S ribosomal RNA sequences database. Best-hit results were used to assign a taxonomy label to isolates.

### 2.8. Statistical Analysis

Analysis of variance (ANOVA) was applied to microbial counts and significant differences were determined using the least significant difference (LSD) test (*p* < 0.05). Differential abundance of taxa was tested by the Mann–Whitney (or Kruskal–Wallis) non-parametric test on relative abundance of taxa (total sum scale). After Kruskal–Wallis, Conover’s test with the FDR Benjamini–Hochberg correction was added for pairwise comparison. Alpha-diversity comparisons were performed using Kruskal–Wallis non-parametric test. Beta-diversity distance matrices and ASV tables were used to calculate principal coordinates (PCoA) and construct ordination plots. The analysis of differential relative abundances of COGs was performed with the Kruskal–Wallis test. The FDR Benjamini–Hochberg correction was used to correct for multiple comparisons. The R packages Biodiversity R version 2.11-1, PMCMR version 4.3, RVAideMemoire version 0.9-7, vegan version 2.5-5, and ‘pheatmap’ version 1.0.12 of R software package version 3.6.0 (http://www.R-project.org, accessed on 10 May 2023) were used.

## 3. Results and Discussion

### 3.1. Analysis of Biofilms by SEM

**Figure 2:**
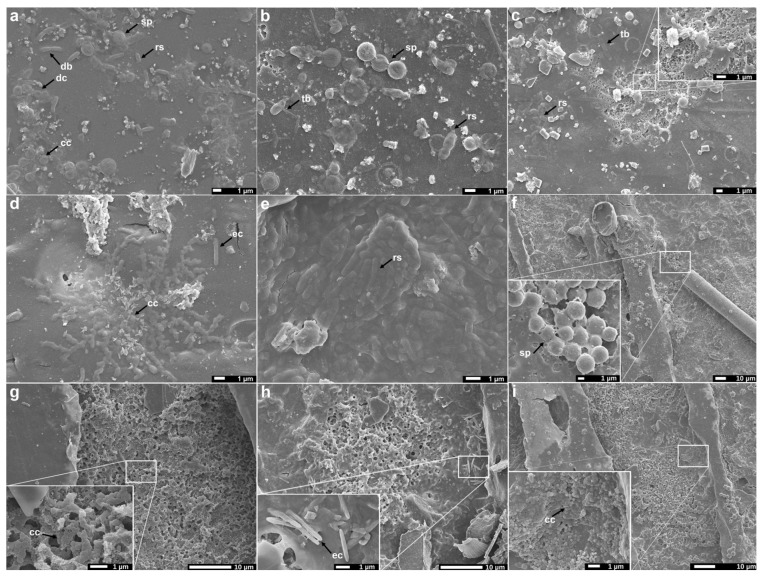
Scanning electron microscopy images of PLA (**a**–**c**) and PHBV (**d**–**i**) films exposed to a marine environment for 6 months. Arrows indicate identified microbial structures. cc: Coccoid cell; db: diplobacilli; ec: elongated cell; rs: rod shape; sp: sporous; tb: tubular.

Biofilms are mini-ecosystems composed of complex communities of microorganisms, which may include bacteria, archaea, eukaryotic microorganisms and microscopic animals, surrounded by a matrix of extrapolymeric substances (EPSs) whose function is to facilitate cell adhesion while offering protection [5,31,32,33,34]. Figure 2 shows the surface of plastic samples after a 6-month exposure. The most prevalent microbial structures in the biofilms were rod-shape (rs) (Figure 2a–c,e) and coccoid (cc) (Figure 2a,d,g,i) cells, which likely corresponded to bacilli and cocci bacteria and, to a lesser extent, to yeasts. Elongated cells (ec) (Figure 2d,h) and fungal spores (sp) (Figure 2b,f) were also observed, together with tubular structures (tb) (Figure 2b,c), which might be fungal hyphae [33]. In some cases, cells seemed to be embedded within the biofilm and surrounded by EPSs (Figure 2d,e). Samples showed extensive areas covered by dense layers of three-dimensional EPSs, especially the PHBV-based films (Figure 2e,g,i), which agrees with our results in terms of microbial biofilm quantification (Section 3.2). On PLA samples, cells tended to be individually distributed or forming smaller aggregates (Figure 2a–c), whereas PHBV samples showed a rougher surface with evident pits and cracks, probably because of biological degradation processes (Figure 2e–i). Similar microbial structures and typical distributions have been widely described by other authors studying marine biofilms formed on petroleum-based macro- and microplastics surfaces [33,35,36,37]. Studies focused on bio-based plastics are scarcer and, to our knowledge, no in-depth comparisons between the two most relevant biopolymers (PLA and PHBV) have been carried out previously. For instance, Zhang et al. (2021) studied PLA biofilms, but they compared them with those formed on conventional plastics, reporting no significant differences.

**Figure 3:**
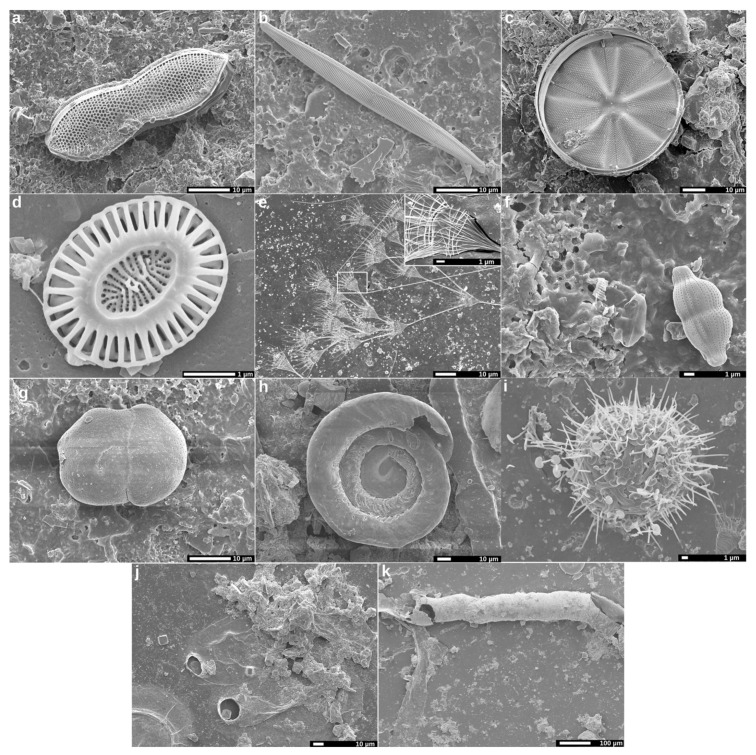
Scanning electron microscopy images showing examples of organisms visualized on PLA (**a**,**e**,**i**,**k**) and PHBV (**b**–**d**,**f**–**h**,**j**,**k**) films exposed to marine environment for 6 months, including pennate and radial diatoms (**a**–**c**), cocolitophores (**d**: *Emiliania huxleyi*), unicellular and colonial choanoflagellates (**e**: *Acanthoeca spectabilis*), foraminifera (**f**–**h**), unidentified structures (**i**), bryozoans (**j**) and polychaete tubs (**k**).

Diatoms were also among the most abundant organisms colonizing samples, as they are considerable on plastic debris [38]. Examples of pennate and centric diatoms, presumably of the *Nitzschia* and *Actinoptychus* genera, are depicted in Figure 3a–c. These microalgae are considered a key factor in biofilm formation, as they produce EPSs (mucilaginous polysaccharides) for their own adhesion [38,39,40]. Additionally, these EPSs could facilitate other organisms’ attachment to plastic surfaces. It is also thought that the production of other extracellular compounds, particularly dissolved organic carbon, could help bacterial development [40]. Moreover, diatoms may be an important food source for invertebrate grazers which, in turn, might exert an indirect physical degradation of the materials [37,41]. Other unicellular algae detected were coccolithophores (Figure 3d), presumably belonging to *Emiliania huxleyi* species [37,42]. The main hypothesis is that coccolithophores would use plastic merely as a physical support [37]. Other biological structures frequently recognized were choanoflagellates (Figure 3e), probably belonging to the *Acanthoeca spectabilis* species, one of the most common choanoflagellates found in marine biofilms [43,44]. These organisms are aquatic bacterivorous filter feeders, which by the presence of flagella create water currents to feed themselves [39,43]. The incidence of foraminifera (Figure 3f–h), bryozoan (Figure 3j) and polychaete tubes (Figure 3k) was also quite frequent. The presence of regular and extended linear grooves found on some samples, mainly on those from PHBV, suggests the occasional occurrence of grazers, which could cause increased physical deterioration on plastic surfaces. These grooves create cavities that could have favored the accumulation of bacterial degraders, promoting bioplastic biodegradation. No polymer-dependent preferential specificity was observed for the diatoms, coccolithophores, choanoflagellates, foraminifera and bryozoans since all of them were found indiscriminately on PLA and PHBV samples at this stage of biofilm formation. This could be attributed to the level of maturity of the studied biofilms, since it has been reported that microbial communities across different surfaces converge as biofilms mature, resulting in minimal differences [45].

### 3.2. Quantification of Microbial Colonization and Dynamics

After 1 month, biofilm formation was visually confirmed, especially on PHBV samples (Figure S1b). As exposure time progressed, the biofilm became thicker and more homogeneously distributed. By the end of the experiment, all samples were covered by an abundant layer of organic matter (Figure S1c,f).

**Figure 4:**
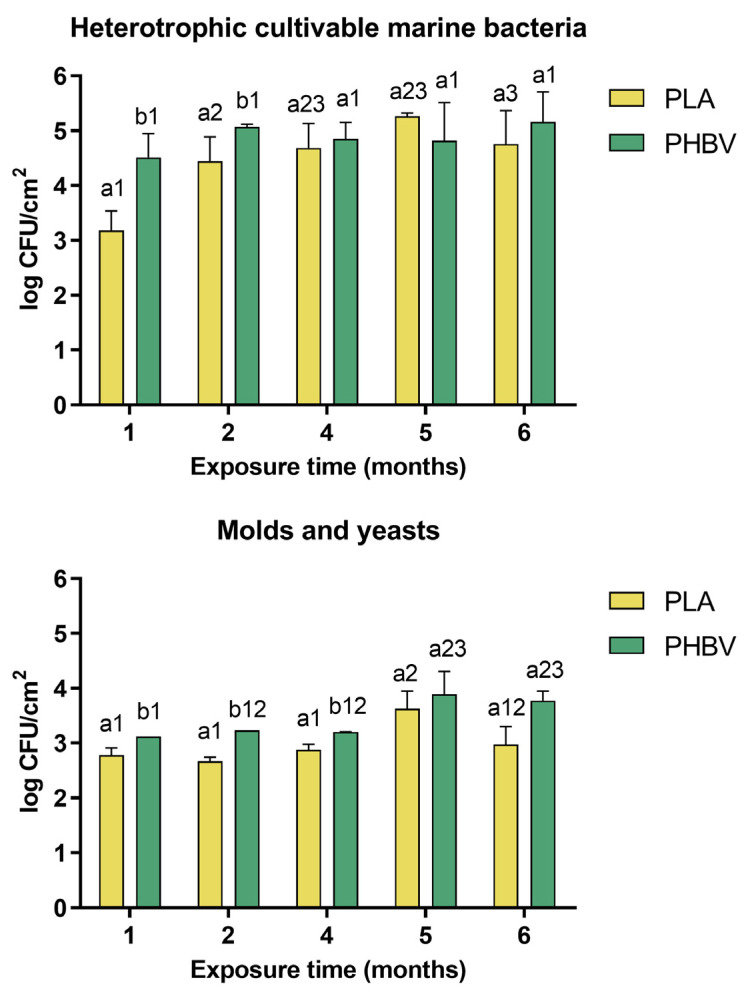
Quantification of marine heterotrophic cultivable bacteria, molds and yeasts on PHBV and PLA samples at different times of exposure. Different letters (a, b) within the same exposure time indicate significant differences (*p* < 0.05) due to type of polymer, and different numbers (1–3) within the same polymer indicate significant differences over time according to the least significant difference (LSD) test.

To quantify the growth of biofilms on PLA and PHBV over time, samples were taken every month and quantification of heterotrophic cultivable bacteria, molds and yeasts was performed (Figure 4). After 1 month of exposure, the culturable bacteria CFUs/cm^2^ significantly differed from one material to another (*p* < 0.05). PHBV samples presented a bacterial surface density (BSD) of 4.51 log CFU/cm^2^, more than one logarithmic unit higher than on PLA, which had 3.18 log CFU/cm^2^. A similar trend was observed for molds and yeasts, with values of 4.12 and 2.77 log CFU/cm^2^ for PHBV and PLA, respectively. A significant increase (*p* < 0.05) in bacterial population took place after 2 months on PLA samples, where the BSD showed a more than one log-fold increase. In the case of PHBV, variations in the BSD over time were not statistically significant (*p* > 0.05). From that point on, the bacterial population size remained stable and only small variations were observed. By the end of the experiment, according to statistical analysis, differences between the materials were not significant (*p* > 0.05), although PHBV samples tended to have higher population sizes, with a final BSD of 5.16 log CFU/cm^2^. Less variation was observed in fungal population sizes. Higher CFU/cm^2^ was observed on PHBV than on PLA biofilms during the first 4 months, and no differences were observed at the longest exposure times. Bacteria CFUs were higher than mold and yeast CFUs at every sampled time point, revealing that bacteria constitute the predominant taxa in the biofilms on both biopolymers, as previously reported [32]. This is consistent with the fact that fungi are less often found in aquatic systems [7]. Observational and quantitative results obtained from 6 months of exposure agree with those already shown for microplastics and bioplastics in freshwater and marine conditions in laboratory experiments [25,46,47]. It is important to acknowledge that culture-dependent techniques, such as plate counts, can only quantify the strains that are capable of being cultured. As a result, these results may not accurately represent the actual number of microorganisms present in the biofilms. However, they do provide a means to compare different samples and draw comparisons between them.

It can be concluded that PHBV provided a more favorable support for further attachment of microorganisms than PLA. In this sense, PHBV would not be only a physical support matrix but also a potential source of nutrients for bacteria, which would favor their multiplication [47]. This is the first time differential results between PLA and PHBV have been demonstrated in situ, since up to now, only comparations between one or the other and conventional plastics have been carried out, and mainly using laboratory closed systems [47].

### 3.3. Analysis of Biofilm Diversity and Composition by Amplicon Sequencing

To gain a deeper insight into the bacterial diversity and taxonomic and functional profiles of the PHBV and PLA biofilms, amplicon sequencing of the V3-V4 regions of the 16S rRNA gene was used. A total of 4522 phylotypes were detected and alpha-diversity analysis revealed that no significant differences were observed for richness and evenness between PHBV and PLA biofilms (Table 1). However, PLA biofilms showed a trend towards higher values for both metrics, which would indicate a higher diversity and uniformity.

**Figure 5:**
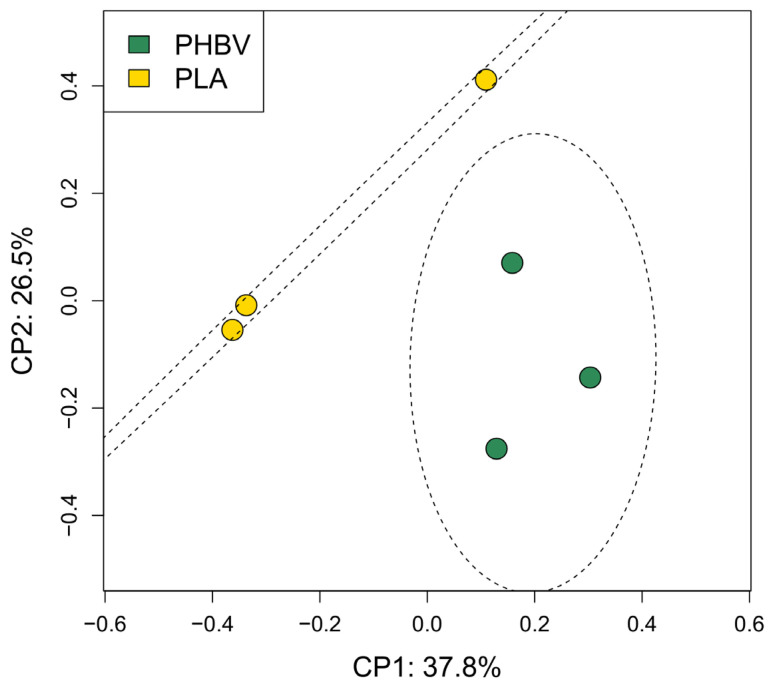
Principal coordinates (PCoA) based on unweighted Unifrac distances showing variation in community structures of PLA and PHBV biofilms formed after 6 months of exposure.

The structure of a biofilm’s bacterial communities was visualized in terms of beta diversity by principal coordinate analysis (PCoA) based on unweighted Unifrac distances (Figure 5). Significant differences between PHBV and PLA biofilms’ communities were observed (Mann–Whitney test, *p* < 0.05) (Figure S2). Unweighted Unifrac distance is based not only on shared phylotypes, but also on the phylogenetic distances between them, indicating putative genomic and functional variation between PLA and PHBV biofilms. In agreement with this, a deep look into the distribution of phylotypes showed that PLA and PHBV biofilms shared 142 phylotypes (there being 2549 phylotypes exclusively found in PLA biofilms and 1652 in those on PHBV) (Figure S3).

**Figure 6:**
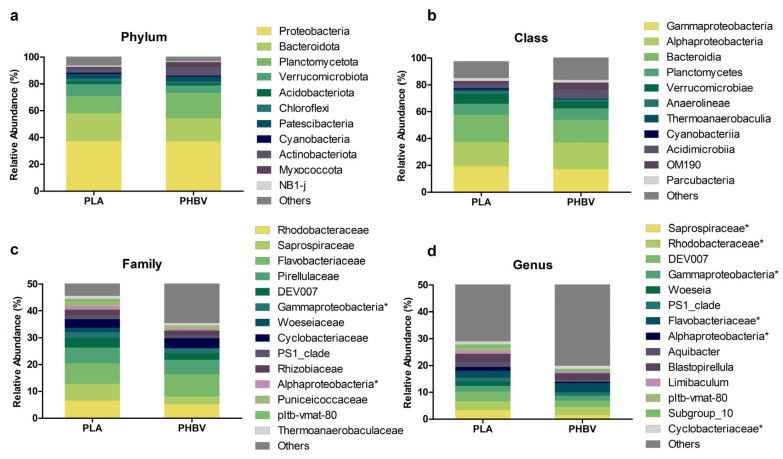
Relative abundances of taxa at the phylum (**a**), class (**b**), family (**c**) and genus (**d**) levels present in PLA and PHBV biofilms. Less abundant taxa (relative abundance lower than 1%) were grouped into the category “Others”. An asterisk (*) indicates “unassigned”.

Taxonomic classification of phylotypes showed that over 70% of the observed bacteria were affiliated to three phyla (Figure 6a). Proteobacteria was the most abundant phylum, followed by Bacteroidota and Planctomycetota. These taxa have already been described among the most frequent phyla colonizing substrates in marine ecosystems [48,49,50,51]. Statistical analysis revealed a significantly higher abundance of Bacteroidota reads on PLA samples, while no significant differences between materials were observed for Proteobacteria and Planctomycetota. Verrucomicrobiota, Acidobacteriota and Chloroflexi were also observed in both PLA and PHA, with a relative abundance value equal to or lower than 10%. Within the Proteobacteria phylum, the Gammaproteobacteria and Alphaproteobacteria classes displayed the highest relative abundances (Figure 6b). Gammaproteobacteria (19%) dominated in PLA biofilms and Alphaproteobacteria was the most prevalent in PHBV samples (20%). Within the Bacteriodota phylum, the Bacteroidia class was the most abundant, being significantly more abundant in PLA biofilms. Planctomycetes was the dominant class within Planctomycetota. It has been previously reported that Alpha- and Gammaproteobacteria are pioneer colonizers on non-biopolymer plastics in marine systems, while Bacteroidia have been considered as secondary colonizers [52,53]. Several authors have reported that Bacteroidota and Proteobacteria, specifically the Gammaproteobacteria and Alphaproteobacteria classes, are the main phyla in the biofilms of floating plastics in the Atlantic and Mediterranean Seas [28,50]. As regards biopolymers-based plastics, many descriptions of in vitro biofilms have been reported. For instance, [47] observed a predominance of Gammaproteobacteria and Alphaproteobacteria in the first and later stages of biofilm development on polyethylene and PHBV in open-circuit aquariums with natural seawater, and found that the community structure of PHBV biofilms significantly differed from the petroleum-based polymer types. For their part, [25] described Proteobacteria and Bacteroidota as the dominant phyla in PHBH biofilms in laboratory freshwater experiments. Our results are in line with these observations, while also being, to our knowledge, the first time significant differences between PLA and PHBV have been reported.

At the family level, Flavobacteriaceae (Bacteroidia) (7.6 and 8.3% on PLA and PHBV), Rhodobacteraceae (Alphaproteobacteria) (6.6 and 5.2% for PLA and PHBV) and Pirellulaceae (Planctomycetes) (5.9 and 5.4% for PLA and PHBV), were the most abundant taxa (Figure 6c). Overall, no statistically significant differences (*p* > 0.05) were observed for these families between polymers. Several researchers have previously reported high abundances of these families on a variety of biofilms grown on marine plastics in different locations [19,25,44,49,50,51,54,55]. The Saprospiraceae (Bacteroidia) family relative abundance was found to differ between PLA and PHBV (6.3 vs. 2.9%). Interestingly, this group is known to comprise members capable of the hydrolysis and utilization of complex organic carbon compounds. Within Saprospiraceae, Portibacter was more abundant in PLA biofilms than in those on PHBV. Even if poorly characterized, this taxon has been previously reported to colonize polyethylene, polyethylene terephthalate and PLA in the marine plastisphere [56,57,58].

The most abundant genus on PLA samples was the uncultured group DEV007 (Verrucomicrobiota), whose abundance (3.6%) was significantly higher than on PHBV (Figure 6d). There are no previous reports of the association of this genus to plastics, although it is known to be present in seawater, attached to nutrient-rich particles [59]. A non-cultured Flavobacteriaceae genus was most abundant on PHBV samples (3.5%). Other relevant genera were uncultured members of the Saprospiraceae and Rhodobacteriaceae families, Blastopirellula (Pirellulaceae) and Aquibacter (Flavobacteriaceae). The presence of the genera Blastopirellula and Aquibacter has been also described on plastic biofilms [54,55].

Bacterial groups with anaerobic and fermentative metabolisms were detected, suggesting that anoxic conditions could occur within the biofilm at some point. One example is the Anaerolineae group (Chloroflexi phylum), comprising 2 and 1% of sequences for PLA and PHBV, respectively. Anaerolineae members have been reported to be associated to sponges and marine sediments [60,61], and they were also detected on plastic debris collected in the North Atlantic [44]. Other fermentative microorganisms observed in biofilms were Parcubacteria, with 2% of relative abundance, and Latescibacteria and Desulfobacteria, within the less abundant groups.

### 3.4. Inference of PLA and PHBV Biofilms’ Metagenomes

So far, the results have agreed with previous observations on marine biofilms: they are highly diverse and complex systems formed by a succession of microbial populations. Differences between PLA and PHBV microbial profiles were detected in the community structures and in individual taxa abundances even at the phylum level. These differences may be reflecting variation in the metabolic abilities of the biofilms. To try to demonstrate this, metagenome functions were predicted by placing phylotypes into a reference tree, which was used as the basis for functional predictions [62]. The functional annotations were based on the COGs database (COG) and 4207 functions (i.e., clusters) were detected.

**Figure 7:**
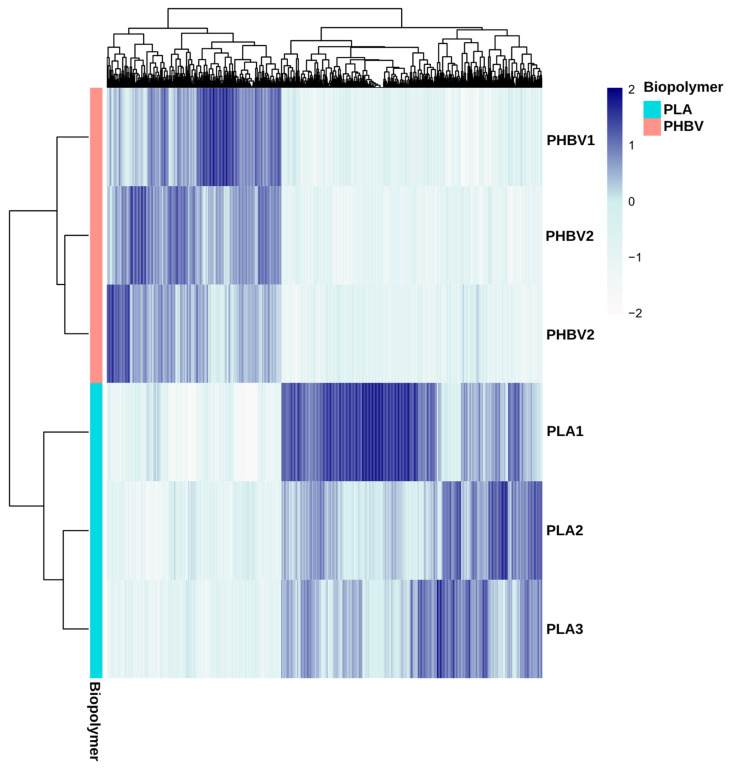
Heatmap of the clusters of orthologous groups (COGs) which present differential relative abundances in the metagenomes of the biofilms formed on PLA and PHBV samples.

A comparison of the predicted metagenomes of PLA and PHBV biofilms showed differences in the relative abundance of 1066 functions. Taking these differences into account, the PLA and PHBV samples clustered apart, revealing distinct functional patterns (Figure 7). Within the significantly different COGs, it stood out that the Poly(3-hydroxybutyrate) depolymerase cluster was more abundant in the PHBV predicted metagenomes. COGs comprising lysophospholipase, dienelactone hydrolase and a predicted esterase of the alpha/beta hydrolase fold were also more abundant in PHBV samples (Table 2). These are part of the alpha/beta-hydrolases superfamily and may also present depolymerase activity [63,64]. Additionally, the COGs related with the patatin-like phospholipase/acyl hydrolase functions were also more abundant in PHBV samples. These proteins have been described in PHB granules of *Cupriavidus necator* where it is encoded in an operon involved in poly-3-hydroxybutyrate utilization [65]. *C. necator* is a well-known bacteria for the production of PHAs [65,66], which would explain the presence in its genome of proteins involved in PHBV metabolization. In addition, COGs with thioesterase activity and other carboxylesterases were also more abundant in PHBV metagenomes, which agrees with hydroxybutyrate metabolism after depolymerization. Thioesterases transform 3-HB in 3-HB-CoA, which can be used later through beta oxidation for the synthesis of acetyl-CoA [67,68]. The Acyl-ACP thioesterase COGs was more abundant in PLA metagenomes (Table 2). Active sites of bacterial thioesterases are known to resemble those of serine proteases, which have been identified as enzymes with PLA-degrading activities [69]. COG1647 and COG4947 related to lipase and esterase functions were also more abundant in PLA samples. Even if these functions are not specific, their presence implies PLA degradation ability in these biofilms. Polyhydroxyalkanoate synthesis regulator phasin COGs were observed in both the PLA and PHBV predicted metagenomes, adding evidence of the presence of polyester plastic metabolism.

### 3.5. Screening of Biopolymer Degraders

**Figure 8:**
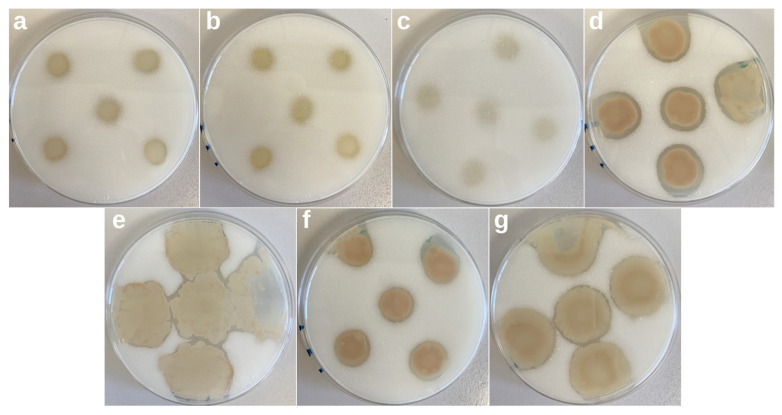
Bottom side of marine agar supplemented with PHBV plates and showing the presence of clearance zones incubated at room temperature for 14 days and inoculated with *Bacillus megaterium* (**a**), *Bacillus sphaericus* (**b**), *Pseudomonas fluorescens* (**c**), strain A11 (**d**), strain B1 (**e**), strain B9 (**f**) and strain C2 (**g**).

Up to 50 bacteria, 18 yeasts and 25 fungi isolates were obtained from the PHBV and PLA samples. Figure S4 shows an example of the top and bottom sides of MA plates amended with PHBV inoculated with isolated bacteria and incubated at room temperature for 10 days. Four bacterial isolates showed evident clear zones around them, revealing their ability to depolymerize PHBV (strains A11, B1, B9 and C2), at least under the tested conditions. Of the known strains, three revealed PHBV-degrading activity: *Bacillus sphaericus*, *Bacillus megaterium* and *Pseudomonas fluorescens*. Figure 8 shows the bottom part of the plates inoculated with the individual strains and showing PHBV-degrading activity. The four bacterial isolates started showing clear zones at the third day of incubation (data not shown), which greatly increased in area and transparency during the following days. In the case of *B. sphaericus*, *B. megaterium* and *P. fluorescens*, the appearance of clear halos took longer, and by the end of the experiment, the clear zones were much less obvious. These results indicate that the environmental isolates possess a more efficient metabolism to biodegrade PHBV and that the isolated bacteria could potentially be responsible for PHBV biodegradation. No clearing zones were observed around yeasts and fungi, indicating that these particular isolates lack PHBV-degradative abilities. In the case of the screening assays for the detection of PLA degraders, neither bacteria, nor yeasts nor fungi led to the appearance of clearance zones on the plates, confirming the limited biodegradability of this polymer in marine environments.

SEM micrographs together with microbial count data, and the results of the in vitro clear-zone method together with the detection of genes encoding proteins related to PHBV biodegradation, corroborate the presence of bacterial PHBV-degrading species in this specific coastal zone environment. Therefore, it can be concluded that, when introduced into seawater, PHBV pieces are enriched with a wide variety of microorganisms, among which some specific bacteria start to biodegrade the polymer.

### 3.6. Molecular Identification of Biopolymer Degraders

Isolates showing PHBV-degrading activity were identified on the basis of their 16S rRNA gene sequences (Table 3). Isolates A11, B1, B9 and C2 were identified as *Vibrio kanaloae*, *Pseudoalteromonas hodoensis*, *Microbulbifer agarilyticus* and *Ruegeria atlantica*, respectively. Similarities between the BLAST query and hit were greater than 99.5% in all cases. The four species identified are members of the Proteobacteria phylum, revealing the importance of this particular bacterial group in the degradation of PHBV. To our knowledge, this is the first time some of these specific species are reported as degraders of PHBV.

V. kanaloae is a relevant plant pathogen [70]. Despite the pathogenicity of some species, *Vibrio* spp. (Gammaproteobacteria) are known to have an active role in the mineralization of organic sediments and other biodegradable materials thanks to their arsenal of extracellular hydrolytic enzymes. In fact, *Vibrio* strains have been reported to degrade polyethylene plastic blends [71]. For its part, the genus *Pseudoalteromonas* (Gammaproteobacteria), has been described as a hydrocarbon degrader and is frequently detected in plastic residues and in association with algae [50,72]. Specifically, *P. hodoensis* has been studied for a wide variety of enzyme production [73]. Similarly, the genus *Microbulbifer* (Gammaproteobacteria) has been of interest due to the production of enzymes related to the degradation of polysaccharides present in algal cell walls [74]. Moreover, species of this genus have been recently reported as plastic degraders [75,76]. Lastly, members of *Ruegeria* sp. (Alphaproteobacteria) have been isolated from various marine environments, and some species, such as *R. lutea*, have been reported to accumulate on PHB [77].

The 16S rRNA sequences from the isolates were compared with the sequences of the biofilm-derived phylotypes (Table S1). BLAST results showed that the best hit for isolate A11 was a phylotype classified within the genus *Vibrio* with 97.65% similarity. No species-level classification was achieved for this phylotype. Isolate B1′s best hit was a phylotype classified within genus *Pseudoalteromonas* with 95.77% similarity. The best hit for isolate B9 was a phylotype assigned to an uncultured species of genus *Eionea* with 90.70% similarity. The best hit (100% similarity) for isolate C2 was classified within genus *Ruegeria*. Moreover, no phylotype was classified as *V. kanaloae*, *P. hodoensis*, *M. agarilyticus* nor *R. atlantica*. These differences in taxonomy assignation were expected since isolate sequences had a mean length of 730 bp while phylotypes had a 413.72 bp mean length. Higher sensitivity in taxonomy classification is achieved with longer sequences. Phylotypes classified as *Vibrio* were present in one PLA and one PHBV sample. The same pattern was observed for *Pseudoalteromonas* phylotypes. As regards *Ruegeria* phylotypes, they were present in all samples. Isolate B9 was not detected in the biofilm dataset since it did not get a best hit with over 95% similarity.

## 4. Conclusions

To the best of our knowledge, this is the first study comparing the microbiological aspects of the biodegradation of PLA and PHBV in real marine conditions. Based on our observations, it can be concluded that bacteria were the predominant microorganisms in the biofilms that formed on the surfaces of the bioplastics, and they were probably those most responsible for PHBV degradation. Other microorganisms present in the biofilms, however, could also indirectly participate in the overall process. After 6 months of exposure, biofilms on PLA and PHBV shared a core microbiome, but significant differences were observed between the polymers which suggested an effect of the biopolymer type on the bacterial community composition and that colonization could be substrate-specific. The presence of specific PHBV degraders, none of which had been previously described as such, was confirmed, and this opened the possibility of further research in this field. The understanding of the microbial diversity at this stage establishes a baseline to comprehend the microbiological aspects behind the biodegradation of biopolymers in marine conditions. However, considering that this work focuses on a relatively short period of exposure, longer exposure times would be required to perform more in-depth studies aimed at assessing the evolution over time of the biofilms’ composition. Moreover, better insight into the interactions between microorganisms in general, and specifically into degraders for these materials, will help to develop efficient approaches to a biopolymer’s end-life disposal.

## Figures and Tables

**Table 1:** Median values for alpha-diversity metrics and Kruskal–Wallis non-parametric test *p* values. *p* values < 0.05 indicate significant differences between the compared experimental groups.

	Observed OTUs	Pielou’s Evenness Index
PLA	1231	0.93
PHBV	693	0.92
*p* value	0.28	0.05

**Table 2:** Differential clusters of orthologous groups (COGs) of proteins involved in PHBV and PLA depolymerization showing significantly different relative abundances in biofilms formed on PHBA and PLA samples.

COG	Description	Relative Abundance	
		PLA	PHBV
**COG0824**	Acyl-CoA thioesterase FadM	1.42 × 10^−3^	1.50 × 10^−3^
**COG0412**	Dienelactone hydrolase	6.80 × 10^−4^	7.98 × 10^−4^
**COG2267**	Lysophospholipase, alpha-beta hydrolase superfamily	6.79 × 10^−4^	7.87 × 10^−4^
**COG1946**	Acyl-CoA thioesterase	2.14 × 10^−4^	3.01 × 10^−4^
**COG3509**	Poly(3-hydroxybutyrate) depolymerase	1.40 × 10^−4^	1.73 × 10^−4^
**COG2272**	Carboxylesterase type B	3.68 × 10^−5^	7.14 × 10^−5^
**COG3320**	Thioester reductase domain of alpha aminoadipate reductase Lys2 and NRPSs	2.70 × 10^−5^	5.49 × 10^−5^
**COG5496**	Predicted thioesterase	2.60 × 10^−5^	4.40 × 10^−5^
**COG3545**	Predicted esterase of the alpha/beta hydrolase fold	2.24 × 10^−5^	3.81 × 10^−5^
**COG3621**	Patatin-like phospholipase/acyl hydrolase	9.43 × 10^−7^	7.12 × 10^−6^
**COG1647**	Esterase/lipase	3.35 × 10^−5^	2.91 × 10^−5^
**COG4947**	Esterase/lipase superfamily enzyme	3.04 × 10^−5^	1.57 × 10^−5^
**COG3884**	Acyl-ACP thioesterase	2.84 × 10^−5^	2.02 × 10^−5^
**COG3937**	Polyhydroxyalkanoate synthesis regulator phasin A	7.12 × 10^−6^	8.98 × 10^−7^

**Table 3:** Identities of the PHBV degraders isolated from the surface of samples exposed to natural marine environment for 6 months.

Isolate	Identification	Maximum Identity (%)	NCBI Database (Accession Number)
**A11**	*Vibrio kanaloae*	100.0	NR_042468.1
**B1**	*Pseudoalteromonas hodoensis*	99.9	NR_126232.1
**B9**	*Microbulbifer agarilyticus*	99.5	NR_041001.1
**C2**	*Ruegeria atlantica*	99.7	NR_112615.1

## Data Availability

The data presented in this study are available on request from the corresponding author.

## Supplementary Materials

The following supporting information can be downloaded at: https://www.mdpi.com/article/10.3390/microorganisms11061461/s1, Figure S1: Visual aspect of PHBV (a–c) and PLA (d–f) samples after 0, 1 and 6 months of exposure to a real marine environment.; Figure S2: Unweighted unifrac distances within PHVB and between PLA and PHBV microbial communities. Mann–Whitney test results show significant differences (*p* < 0.05); Figure S3: Venn diagram showing shared and specific operational taxonomic units (OTUs) present in the biofilms formed on PLA and PHBV samples; Figure S4: Top and bottom side of marine agar supplemented with PHBV plates used for the screening of PHBV-degrading microbes; Table S1: BLAST results of the comparison between the 16S rRNA gene sequences from the isolates and the representative sequences of the biofilm-derived operational taxonomic units (OTUs).

## Appendix A

ASVs were aligned using the QIIME alignment mafft method [78]. The alignment was used to create a tree and to calculate phylogenetic relations between ASVs using the QIIME phylogeny fasttree method. ASV tables were subsampled without replacement to even sample sizes for diversity analysis using the QIIME diversity core-metrics-phylogenetic pipeline. The smallest sample size was chosen for subsampling. Taxonomic assignment of ASVs was performed using a Bayesian classifier trained with the Silva database (i.e., 99% OTUs database) using the QIIME feature-classifier classify-sklearn method (Wang et al., 2007). Phylotypes were filtered to discard contaminant Eukariota DNA-derived amplicons using the basic local alignment search tool (BLAST) against the mentioned database with a 90% identity cut-off.

## References

[1] Jambeck J., Geyer R., Wilcox C., Siegler T.R., Perryman M., Andrady A., Narayan R., Law K.L. (2015). Plastic waste inputs from land into the ocean. Mar. Pollut..

[2] Briassoulis D., Pikasi A., Briassoulis C., Mistriotis A. (2019). Disintegration behaviour of bio-based plastics in coastal zone marine environments: A field experiment under natural conditions. Sci. Total Environ..

[3] Brunner I., Fischer M., Rüthi J., Stierli B., Frey B. (2018). Ability of fungi isolated from plastic debris floating in the shoreline of a lake to degrade plastics. PLoS ONE.

[4] European B. Nova-Institute Bioplastics Market Data 2018. Global Production Capacities of Bioplastics 2018–2013. https://www.european-bioplastics.org/wp-content/uploads/2016/02/Report_Bioplastics-Market-Data_2018.pdf.

[5] Dilkes-Hoffman L.S., Lant P.A., Laycock B., Pratt S. (2019). The rate of biodegradation of PHA bioplastics in the marine environment: A meta-study. Mar. Pollut. Bull..

[6] European B. Nova-Institute Bioplastics Market Update 2020. https://www.european-bioplastics.org/market-update-2020-bioplastics-continue-to-become-mainstream-as-the-global-bioplastics-market-is-set-to-grow-by-36-percent-over-the-next-5-years/.

[7] Meereboer K.W., Misra M., Mohanty A.K. (2020). Review of recent advances in the biodegradability of polyhydroxyalkanoate (PHA) bioplastics and their composites. Green Chem..

[8] Deroiné M., César G., Le Duigou A., Davies P., Bruzaud S. (2015). Natural Degradation and Biodegradation of Poly(3-Hydroxybutyrate-co-3-Hydroxyvalerate) in Liquid and Solid Marine Environments. J. Polym. Environ..

[9] Karamanlioglu M., Preziosi R., Robson G.D. (2017). Abiotic and biotic environmental degradation of the bioplastic polymer poly(lactic acid): A review. Polym. Degrad. Stab..

[10] Singha S., Hedenqvist M.S. (2020). A review on barrier properties of poly(lactic Acid)/clay nanocomposites. Polymers.

[11] Marano S., Laudadio E., Minnelli C., Stipa P. (2022). Tailoring the Barrier Properties of PLA: A State-of-the-Art Review for Food Packaging Applications. Polymers.

[12] Melendez-Rodriguez B., Torres-Giner S., Angulo I., Pardo-Figuerez M., Hilliou L., Escuin J.M., Cabedo L., Nevo Y., Prieto C., Lagaron J.M. (2021). High-oxygen-barrier multilayer films based on polyhydroxyalkanoates and cellulose nanocrystals. Nanomaterials.

[13] Sanchez-Safont E.L., Gonzalez-Ausejo J., Gamez-Perez J., Lagaron J.M., Cabedo L. (2016). Poly(3-Hydroxybutyrate-co-3-Hydroxyvalerate)/purified cellulose fiber composites by melt blending: Characterization and degradation in composting conditions. J. Renew. Mater..

[14] Volova T.G., Boyandin A.N., Vasil’ev A.D., Karpov V.A., Kozhevnikov I.V., Prudnikova S.V., Rudnev V.P., Xuån B.B., Dũng V.V., Gitel’zon I.I. (2011). Biodegradation of polyhydroxyalkanoates (PHAs) in the South China Sea and identification of PHA-degrading bacteria. Microbiology.

[15] Nakayama A., Yamano N., Kawasaki N. (2019). Biodegradation in seawater of aliphatic polyesters. Polym. Degrad. Stab..

[16] Chamas A., Moon H., Zheng J., Qiu Y., Tabassum T., Jang J.H., Abu-Omar M., Scott S.L., Suh S. (2020). Degradation Rates of Plastics in the Environment. ACS Sustain. Chem. Eng..

[17] Tsuji H., Suzuyoshi K. (2002). Environmental degradation of biodegradable polyesters 2. Poly(ε-caprolactone), poly[(R)-3-hydroxybutyrate], and poly(L-lactide) films in natural dynamic seawater. Polym. Degrad. Stab..

[18] Tsuji H., Suzuyoshi K. (2002). Environmental degradation of biodegradable polyesters and poly (L-lactide) films in controlled static seawater. Polym. Degrad. Stab..

[19] Pinto M., Langer T.M., Hüffer T., Hofmann T., Herndl G.J. (2019). The composition of bacterial communities associated with plastic biofilms differs between different polymers and stages of biofilm succession. PLoS ONE.

[20] Veses V., González-Torres P., Carbonetto B., Jovani-Sancho M.D.M., González-Martínez R., Cortell-Ballester I., Sheth C.C. (2020). Dental black plaque: Metagenomic characterization and comparative analysis with white-plaque. Sci. Rep..

[21] Boylen E., Rideout J.R., Dillon M.R. (2019). Reproducible, interactive, scalable and extensible microbiome data science using QIIME 2. Nat. Biotechnol..

[22] Callahan B.J., McMurdie P.J., Rosen M.J., Han A.W., Johnson A.J.A., Holmes S.P. (2016). DADA2: High-resolution sample inference from Illumina amplicon data. Nat. Methods.

[23] Lozupone C., Lladser M.E., Knights D., Stombaugh J., Knight R. (2011). UniFrac: An effective distance metric for microbial community comparison. ISME J..

[24] Venn Diagram Plotter Tool. https://bioinformatics.psb.ugent.be/webtools/Venn/.

[25] Morohoshi T., Ogata K., Okura T., Sato S. (2018). Molecular characterization of the bacterial community in biofilms for degradation of poly(3-hydroxybutyrate-co-3-hydroxyhexanoate) films in seawater. Microbes Environ..

[26] Volova T.G., Prudnikova S.V., Vinogradova O.N., Syrvacheva D.A., Shishatskaya E.I. (2017). Microbial Degradation of Polyhydroxyalkanoates with Different Chemical Compositions and Their Biodegradability. Microb. Ecol..

[27] (2017). Standard Test Method for Determining Aerobic Biodegradation of Plastic Materials in the Marine Environment by a Defined Microbial Consortium or Natural Sea Water Inoculum.

[28] Delacuvellerie A., Cyriaque V., Gobert S., Benali S., Wattiez R. (2019). The plastisphere in marine ecosystem hosts potential specific microbial degraders including Alcanivorax borkumensis as a key player for the low-density polyethylene degradation. J. Hazard. Mater..

[29] Cafaro V., Izzo V., Notomista E., Di Donato A. (2013). Marine Hydrocarbonoclastic Bacteria.

[30] Lane D.J., Stackebrandt E., Goodfellow M. (1995). 16S/23S rRNA Sequencing. Nucleic Acid Techniques in Bacterial Systematic.

[31] Sosa A., Chen F. (2022). An In Situ Study to Understand Community Structure of Estuarine Microbes on the Plastisphere. Microorganisms.

[32] Salta M., Wharton J.A., Blache Y., Stokes K.R., Briand J.F. (2013). Marine biofilms on artificial surfaces: Structure and dynamics. Environ. Microbiol..

[33] Ramsperger A.F.R.M., Stellwag A.C., Caspari A., Fery A., Lueders T., Kress H., Löder M.G.J., Laforsch C. (2020). Structural diversity in early-stage biofilm formation on microplastics depends on environmental medium and polymer properties. Water.

[34] Shi X., Chen Z., Wei W., Chen J., Ni B.-J. (2023). Toxicity of micro/nanoplastics in the environment: Roles of plastisphere and eco-corona. Soil Environ. Health.

[35] Feng L., He L., Jiang S., Chen J., Zhou C., Qian Z.J., Hong P., Sun S., Li C. (2020). Investigating the composition and distribution of microplastics surface biofilms in coral areas. Chemosphere.

[36] Bryant J.A., Clemente T.M., Viviani D.A., Fong A.A., Thomas K.A., Kemp P., Karl D.M., White A.E., DeLong E.F. (2016). Diversity and Activity of Communities Inhabiting Plastic Debris in the North Pacific Gyre. MSystems.

[37] Reisser J., Shaw J., Hallegraeff G., Proietti M., Barnes D.K.A., Thums M., Wilcox C., Hardesty B.D., Pattiaratchi C. (2014). Millimeter-sized marine plastics: A new pelagic habitat for microorganisms and invertebrates. PLoS ONE.

[38] Du Y., Liu X., Dong X., Yin Z. (2022). A review on marine plastisphere: Biodiversity, formation, and role in degradation. Comput. Struct. Biotechnol. J..

[39] Esensoy F.B., Şentürk Y., Aytan Ü., Ülgen A., Pogojeva M., Simeonova A. (2020). Microbial biofilm on plastics in the southeastern Black Sea. Marine Litter in Black Sea.

[40] Hoagland K.D., Rosowski J.R., Gretz M.R., Roemer S.C. (1993). Diatom extracellular polymeric substances: Function, fine structure, chemistry and physiology. J. Phycol..

[41] Zhang B., Yang X., Liu L., Chen L., Teng J., Zhu X., Zhao J., Wang Q. (2021). Spatial and seasonal variations in biofilm formation on microplastics in coastal waters. Sci. Total Environ..

[42] Masó M., Fortuño J.-M., de Juan S., Demestre M., Pelegrí J.L., Vaqué D. (2016). Microfouling communities from pelagic and benthic marine plastic debris sampled across Mediterranean coastal waters. Scientia Marina.

[43] Leadbeater B.S.C. (2015). The Choanoflagellates: Evolution, Biology and Ecology.

[44] Zettler E.R., Mincer T.J., Amaral-Zettler L.A. (2013). Life in the “plastisphere”: Microbial communities on plastic marine debris. Environ. Sci. Technol..

[45] Zhi Xiang J.K., Bairoliya S., Cho Z.T., Cao B. (2023). Plastic-microbe interaction in the marine environment: Research methods and opportunities. Environ. Int..

[46] Jacquin J., Callac N., Cheng J., Giraud C., Gorand Y., Denoual C., Pujo-Pay M., Conan P., Meistertzheim A.L., Barbe V. (2021). Microbial Diversity and Activity During the Biodegradation in Seawater of Various Substitutes to Conventional Plastic Cotton Swab Sticks. Front. Microbiol..

[47] Dussud C., Hudec C., George M., Fabre P., Higgs P., Bruzaud S., Delort A.M., Eyheraguibel B., Meistertzheim A.L., Jacquin J. (2018). Colonization of non-biodegradable and biodegradable plastics by marine microorganisms. Front. Microbiol..

[48] Tourova T., Sokolova D., Nazina T., Grouzdev D., Kurshev E., Laptev A. (2020). Biodiversity of microorganisms colonizing the surface of polystyrene samples exposed to different aqueous environments. Sustainability.

[49] Roager L., Sonnenschein E.C. (2019). Bacterial Candidates for Colonization and Degradation of Marine Plastic Debris. Environ. Sci. Technol..

[50] Frère L., Maignien L., Chalopin M., Huvet A., Rinnert E., Morrison H., Kerninon S., Cassone A.L., Lambert C., Reveillaud J. (2018). Microplastic bacterial communities in the Bay of Brest: Influence of polymer type and size. Environ. Pollut..

[51] Kallscheuer N., Jogler M., Wiegand S., Peeters S.H., Heuer A., Boedeker C., Jetten M.S.M., Rohde M., Jogler C. (2020). Three novel Rubripirellula species isolated from plastic particles submerged in the Baltic Sea and the estuary of the river Warnow in northern Germany. Antonie Van Leeuwenhoek Int. J. Gen. Mol. Microbiol..

[52] Agostini L., Moreira J.C.F., Bendia A.G., Kmit M.C.P., Waters L.G., Santana M.F.M., Sumida P.Y.G., Turra A., Pellizari V.H. (2021). Deep-sea plastisphere: Long-term colonization by plastic-associated bacterial and archaeal communities in the Southwest Atlantic Ocean. Sci. Total Environ..

[53] Lee J.W., Nam J.H., Kim Y.H., Lee K.H., Lee D.H. (2008). Bacterial communities in the initial stage of marine biofilm formation on artificial surfaces. J. Microbiol..

[54] Kirstein I.V., Wichels A., Gullans E., Krohne G., Gerdts G. (2019). The plastisphere–Uncovering tightly attached plastic “specific” microorganisms. PLoS ONE.

[55] Oberbeckmann S., Kreikemeyer B., Labrenz M. (2018). Environmental factors support the formation of specific bacterial assemblages on microplastics. Front. Microbiol..

[56] Marsay K.S., Koucherov Y., Davidov K., Iankelevich-Kounio E., Itzahri S., Salmon-Divon M., Oren M. (2022). High-resolution screening for marine prokaryotic and eukaryotic taxa with selective preference for PE and PET surfaces. BioRxiv.

[57] Cheng J., Jacquin J., Conan P., Pujo-Pay M., Barbe V., George M., Fabre P., Bruzaud S., Ter Halle A., Meistertzheim A.L. (2021). Relative Influence of Plastic Debris Size and Shape, Chemical Composition and Phytoplankton-Bacteria Interactions in Driving Seawater Plastisphere Abundance, Diversity and Activity. Front. Microbiol..

[58] Amaral-Zettler L.A., Ballerini T., Zettler E.R., Asbun A.A., Adame A., Casotti R., Dumontet B., Donnarumma V., Engelmann J.C., Frère L. (2021). Diversity and predicted inter- and intra-domain interactions in the Mediterranean Plastisphere. Environ. Pollut..

[59] Coskun Ö.K., Özen V., Wankel S.D., Orsi W.D. (2019). Quantifying population-specific growth in benthic bacterial communities under low oxygen using H218O. ISME J..

[60] Cleary D.F.R., Polónia A.R.M., Becking L.E., de Voogd N.J., Purwanto, Gomes H., Gomes N.C.M. (2018). Compositional analysis of bacterial communities in seawater, sediment, and sponges in the Misool coral reef system, Indonesia. Mar. Biodivers..

[61] Blazejak A., Schippers A. (2010). High abundance of JS-1- and Chloroflexi-related Bacteria in deeply buried marine sediments revealed by quantitative, real-time PCR. FEMS Microbiol. Ecol..

[62] Douglas G.M., Maffei V.J., Zaneveld J.R., Yurgel S.N., Brown J.R., Taylor C.M., Huttenhower C., Langille M.G.I. (2020). PICRUSt2 for prediction of metagenome functions. Nat. Biotechnol..

[63] Dimitriou P.S., Denesyuk A.I., Nakayama T., Johnson M.S., Denessiouk K. (2019). Distinctive structural motifs co-ordinate the catalytic nucleophile and the residues of the oxyanion hole in the alpha/beta-hydrolase fold enzymes. Protein Sci..

[64] Oh C., Doohun Kim T., Kim K.K. (2019). Carboxylic ester hydrolases in bacteria: Active site, structure, function and application. Crystals.

[65] Sznajder A., Pfeiffer D., Jendrossek D. (2015). Comparative proteome analysis reveals four novel polyhydroxybutyrate (PHB) granule-associated proteins in Ralstonia eutropha H16. Appl. Environ. Microbiol..

[66] Novackova I., Kucera D., Porizka J., Pernicova I., Sedlacek P., Koller M., Kovalcik A., Obruca S. (2019). Adaptation of Cupriavidus necator to levulinic acid for enhanced production of P(3HB-co-3HV) copolyesters. Biochem. Eng. J..

[67] Wang Q., Yu H., Xia Y., Kang Z., Qi Q. (2009). Complete PHB mobilization in Escherichia coli enhances the stress tolerance: A potential biotechnological application. Microb. Cell Fact..

[68] Cai G.Q., Driscoll B.T., Charles T.C. (2000). Requirement for the enzymes acetoacetyl coenzyme A synthetase and poly- 3-hydroxybutyrate (PHB) synthase for growth of Sinorhizobium meliloti on PHB cycle intermediates. J. Bacteriol..

[69] Butbunchu N., Pathom-Aree W. (2019). Actinobacteria as Promising Candidate for Polylactic Acid Type Bioplastic Degradation. Front. Microbiol..

[70] Huang B., Zhang X., Wang C., Bai C., Li C., Li C., Xin L. (2021). Isolation and characterization of Vibrio harveyi as a major pathogen associated with mass mortalities of ark clam, Scapharca broughtonii, in summer. Aquaculture.

[71] Raghul S.S., Bhat S.G., Chandrasekaran M., Francis V., Thachil E.T. (2014). Biodegradation of polyvinyl alcohol-low linear density polyethylene-blended plastic film by consortium of marine benthic vibrios. Int. J. Environ. Sci. Technol..

[72] Oberbeckmann S., Osborn A.M., Duhaime M.B. (2016). Microbes on a bottle: Substrate, season and geography influence community composition of microbes colonizing marine plastic debris. PLoS ONE.

[73] Li A., Luo H., Hu T., Huang J., Alam N.U., Meng Y., Meng F., Korkor N.L., Hu X., Li O. (2019). Screening and enzymatic activity of high-efficiency gellan lyase producing bacteria Pseudoalteromonas hodoensis PE1. Bioengineered.

[74] Miyazaki M., Nogi Y., Ohta Y., Hatada Y., Fujiwara Y., Ito S., Horikoshi K. (2008). Microbulbifer agarilyticus sp. nov. and Microbulbifer thermotolerans sp. nov., agar-degrading bacteria isolated from deep-sea sediment. Int. J. Syst. Evol. Microbiol..

[75] Park S.L., Cho J.Y., Kim S.H., Bhatia S.K., Gurav R., Park S., Park K., Yang Y. (2021). Isolation of Microbulbider sp. SOL66 with High Polyhydroxyalkanoate-Degrading Activity from the Marine Environment. Polymers.

[76] Li Z., Wei R., Gao M., Ren Y., Yu B., Nie K., Xu H., Liu L. (2020). Biodegradation of low-density polyethylene by Microbulbifer hydrolyticus IRE-31. J. Environ. Manag..

[77] Kim J., Kim D.Y., Yang K.H., Kim S., Lee S.S. (2019). Ruegeria lutea sp. Nov., isolated from marine sediment, Masan Bay, South Korea. Int. J. Syst. Evol. Microbiol..

[78] Katoh K., Standley D.M. (2013). MAFFT Multiple Sequence Alignment Software Version 7: Improvements in Performance and Usability. Mol. Biol. Evol..

