# Supplementary material for: Microbiological Characterization of the Biofilms Colonizing Bioplastics in Natural Marine Conditions: A Comparison between PHBV and PLA
Source: Microorganisms. 2023 May 31;11(6):1461. doi: 10.3390/microorganisms11061461 (PMC10304962; doi:10.3390/microorganisms11061461)
Supplement: Supplementary file 1 [file microorganisms-11-01461-s001.zip › microorganisms-2417798-supplementary.pdf]

Article – Supplementary Material

Microbiological Characterization of the Biofilms Colonizing Bioplastics in Natural Marine Conditions: A Comparison between PHBV and PLA

Anna Marín [1], Patricia Feijoo [1], Rosa de Llanos [2], Belén Carbonetto [3], Pedro González-Torres [3], José Tena-Medialdea [4], José R. García-March [4], José Gámez-Pérez [1] and Luis Cabedo [1*]

Number of pages: 4

Number of figures: 4

Number of tables: 1

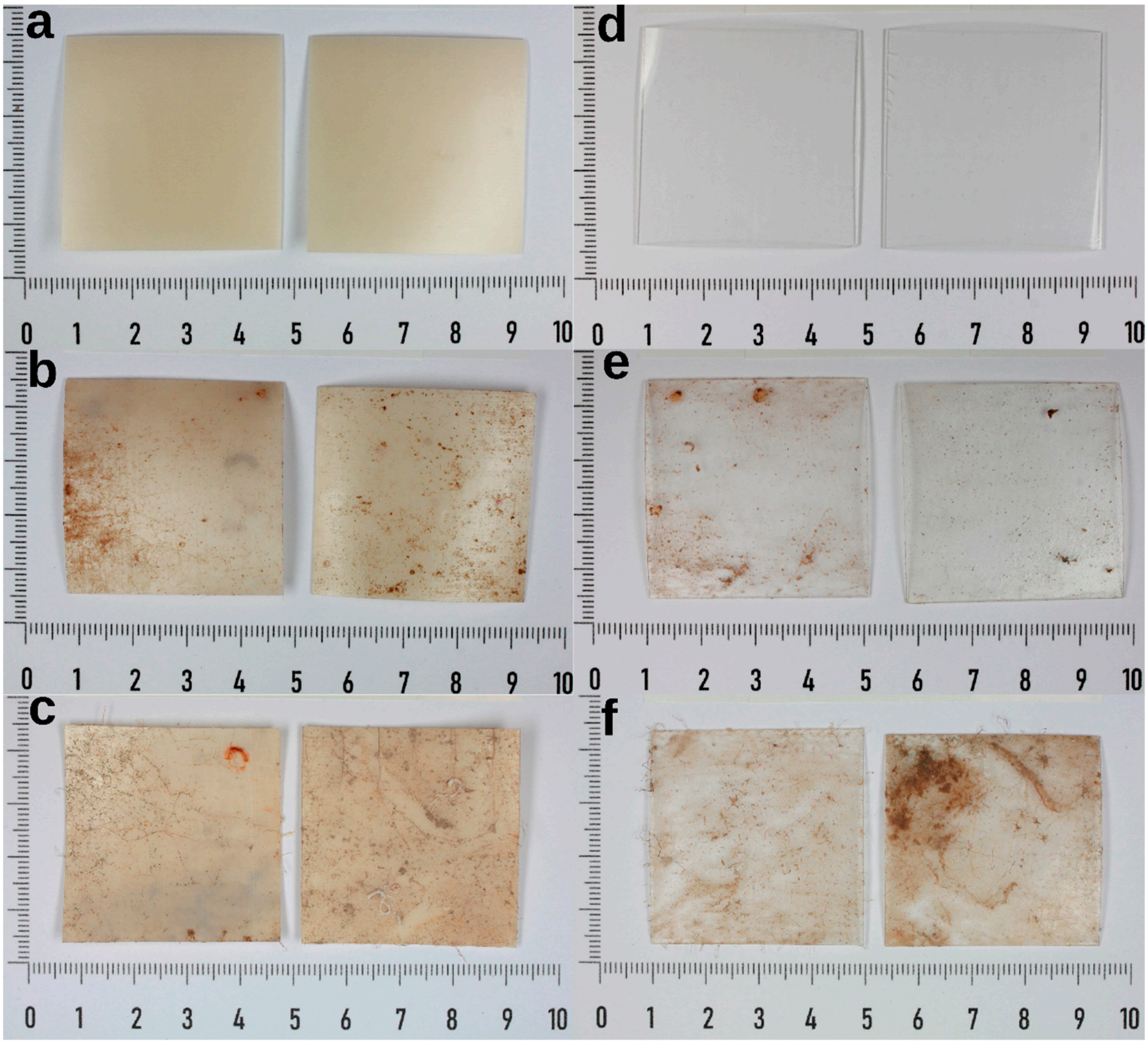

Figure S1. Visual aspect of PHBV (a, b, c) and PLA (d, e, f) samples after 0, 1 and 6 months of exposure to a real marine environment.

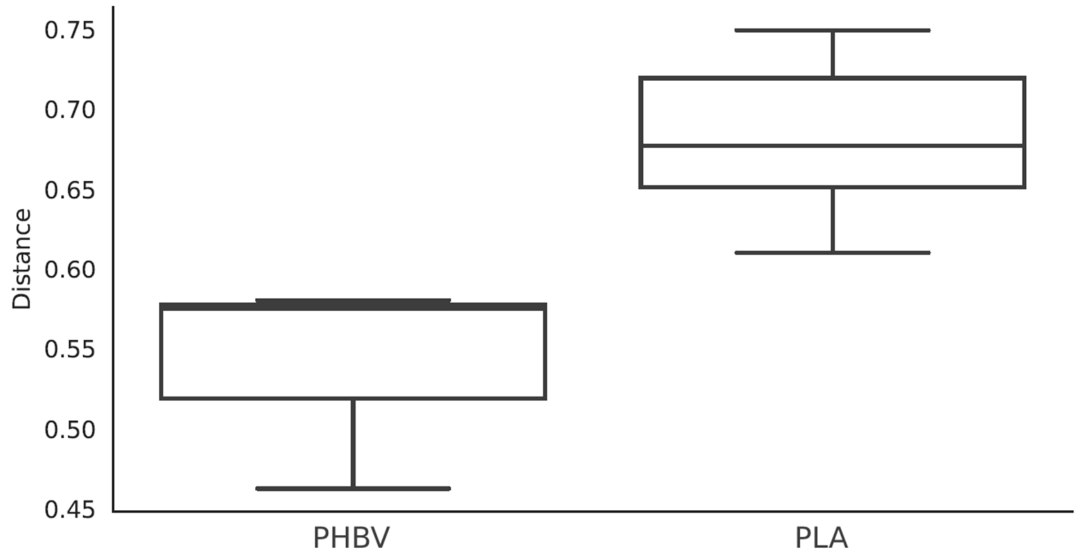

Figure S2. Unweighted Unifrac distances within PHVB and between PLA and PHBV microbial communities. Mann Whitney test results show significant differences ($p < 0.05$).

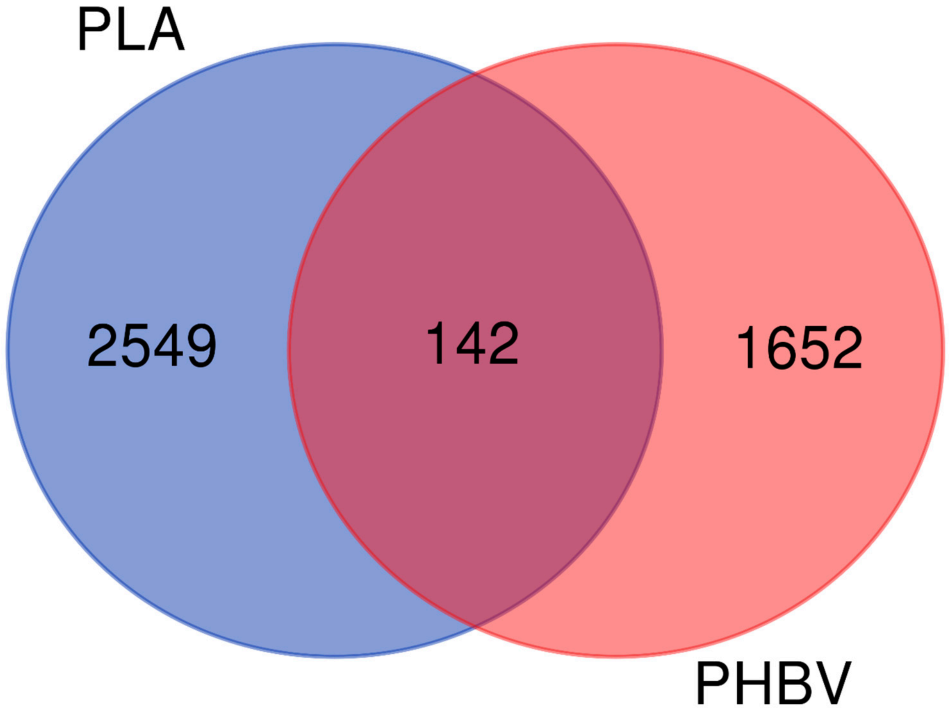

Figure S3. Venn diagram showing shared and specific operational taxonomic units (OTUs) present in the biofilms formed on PLA and PHBV samples.

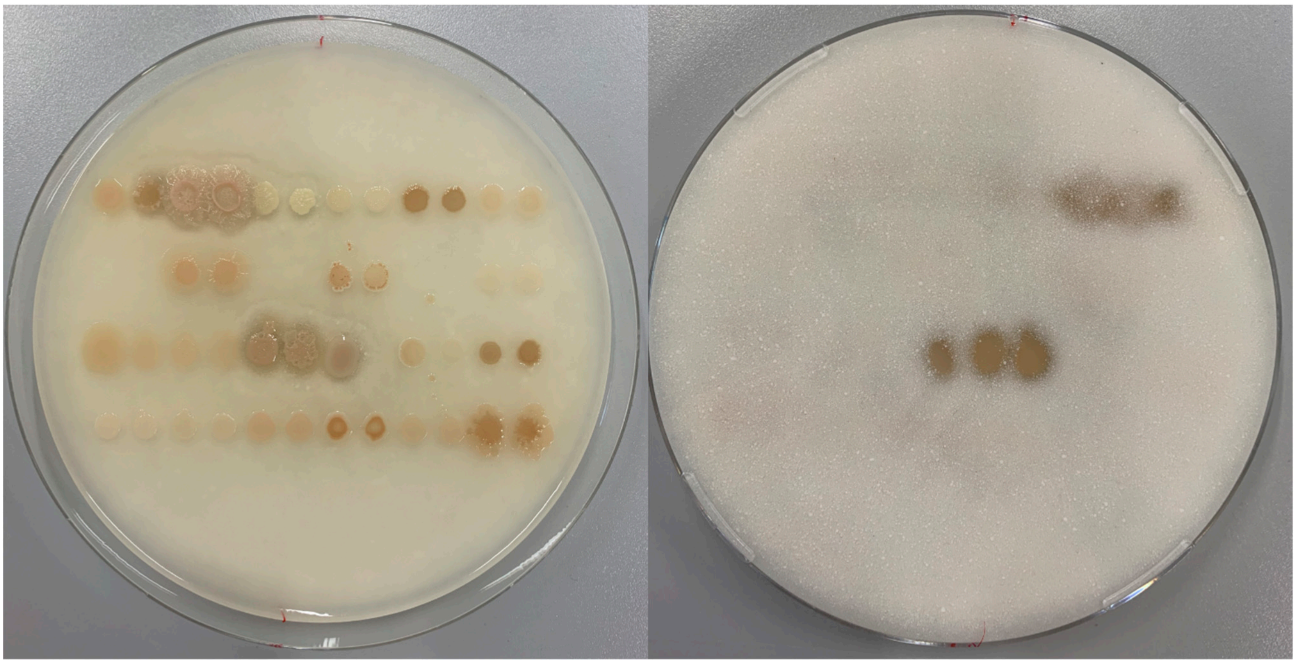

Figure S4. Top and bottom side of marine agar supplemented with PHBV plates used for the screening of PHBV-degrading microbes**.**

Table S1. Blast results of the comparison between the 16S rRNA gene sequences from the isolates and the representative sequences of the biofilm derived operational taxonomic units (OTUs).

Isolate	Phylotype ID	Taxon	% Identity	Alignment lenght	Mismatches	Gap opens	q. start	q.end	s.start	s.end	Evalue	Bit score
**C2**	7d119e9798a893c2d997edbf15545dd0	*Ruegeria*	100	399	0	0	191	399	1	0	399	737
**A11**	1460d734d3638e476053a9a7b1d5099a	*Vibrio*	97.66	428	4	5	86	425	1	0	425	730
**B1**	2dd6b48401ed789955e2cad835e9fbc0	*Pseudoalteromonas*	95.76	425	14	4	169	423	1	0	423	682
**B9**	f24e8d1c913ec880d892ab69508f259d	uncultured_*Eionea*	90.40	427	37	4	116	425	1	8.37-160	425	558
